# Sugar or Fat?—Metabolic Requirements for Immunity to Viral Infections

**DOI:** 10.3389/fimmu.2017.01311

**Published:** 2017-10-16

**Authors:** Hesham M. Shehata, Andrew J. Murphy, Man kit Sam Lee, Clair M. Gardiner, Suzanne M. Crowe, Shomyseh Sanjabi, David K. Finlay, Clovis Steve Palmer

**Affiliations:** ^1^Virology and Immunology, Gladstone Institutes, San Francisco, CA, United States; ^2^Haematopoiesis and Leukocyte Biology, Baker Heart and Diabetes Institute, Melbourne, VIC, Australia; ^3^School of Biochemistry and Immunology, Trinity College Dublin, Trinity Biomedical Sciences Institute, Dublin, Ireland; ^4^Centre for Biomedical Research, Burnet Institute, Melbourne, VIC, Australia; ^5^Department of Infectious Diseases, Monash University, Melbourne, VIC, Australia; ^6^Department of Microbiology and Immunology, University of Melbourne, Melbourne, VIC, Australia

**Keywords:** immunometabolism, glycolysis, mTOR, HIV, T cells, viral infection

## Abstract

The realization that an intricate link exists between the metabolic state of immune cells and the nature of the elicited immune responses has brought a dramatic evolution to the field of immunology. We will focus on how metabolic reprogramming through the use of glycolysis and fatty-acid oxidation (sugar or fat) regulates the capacity of immune cells to mount robust and effective immune responses. We will also discuss how fine-tuning sugar and fat metabolism may be exploited as a novel immunotherapeutic strategy to fight viral infections or improve vaccine efficacy.

## Introduction

In response to an immunological challenge, such as a viral infection, cells of the immune system engage in robust growth and proliferation to generate effector cells that produce large quantities of effector molecules, such as cytokines and cytotoxic granules. Therefore, immune activation is associated with prodigious biosynthetic and energy demands, and immune cells must efficiently and robustly engage in metabolic reprogramming to generate sufficient energy to fuel these demands. Thus, immune cells reprogram cellular metabolic pathways that utilize both sugar and fat to accommodate the increased synthesis of metabolic intermediates required to synthesize cellular components and immunological effector molecules (e.g., amino acids, nucleotides, lipids). Developing a strong understanding of these biosynthetic pathways has immense implications for fine-tuning T cell functions for immunotherapeutic purposes (1).

Metabolic pathways in naïve T cells (Tn) are configured to efficiently convert glucose into energy, in the form of adenosine triphosphate (ATP), which is the main requirement for these relatively quiescent cells. After immune activation, T cells undergo substantial metabolic changes, involving the induction of metabolic genes that result in highly elevated levels of nutrient uptake, including glucose, glutamine, and other amino acids (2–5). Yet, even under aerobic conditions, rapidly proliferating T cells ferment glucose to lactate despite the presence of oxygen (6–8). This metabolic adaptation called the Warburg effect was first described in cancer cells, which utilize glucose predominantly *^via^* glycolysis for growth and survival even in the presence of oxygen (7, 8). The biological reasons underlying this metabolic switch to fermenting glucose to lactate has remained poorly understood since Otto Warburg first described the phenomenon in cancer cells over 90 years ago (9). It has been appreciated that high rates of glucose uptake allow glycolytic intermediates to be used for biosynthetic pathways, such as the pentose phosphate pathway and the one-carbon metabolic pathway for the synthesis of nucleotides and for generating the precursors for lipid and amino-acid biosynthesis required for cell growth and proliferation (9, 10). However, these biosynthetic demands only account for less than 10% of the increased glucose uptake driven by the Warburg effect (11, 12). Thus, it is clear that the increased glucose uptake has other critical functions. Recently, new evidence points that glucose and glutamine can be shunted to the hexosamine biosynthetic pathway (HBP) which controls the production of uridine diphosphate *N*-acetyl glucosamine (UDP-GlcNAc) that is a substrate for protein glycosylation, which has critical downstream implications for T cell clonal expansion and function (13). Thus, the adoption of an activated phenotype from the naïve state is accompanied by elevated glycolysis that fuels the generation of the precursors required for cellular growth and proliferation.

Like activated T cells, monocytes and dendritic cells stimulated with toll-like receptor (TLR) agonists, growth, differentiation, and inflammatory cytokine production rely on glycolysis and produce high levels of lactate. For cells of the myeloid lineage, metabolic analyses have so far focused on those driven by bacterial infections and those critical for monocyte differentiation into inflammatory macrophage subsets (14). In both scenarios, macrophages have been shown to adopt a glycolytic phenotype. This review will focus on metabolic changes that underpin T cell functions. However, we will briefly discuss the role of monocyte and macrophage metabolism in orchestrating systemic inflammation and disease outcomes in chronic viral infections.

We will emphasize the key metabolic pathways that regulate the development and function of T cells during their response to acute and chronic infections. In particular, we will focus on how metabolic reprogramming orchestrates the cell fate decision and functions of CD8^+^ T cells. We will also discuss in detail how metabolism alters CD8^+^, CD4^+^ T cell, and monocyte functions in the context of HBV and HIV infection. Relevant to this discussion are the recent developments in the field to manipulate immunometabolism as an attractive target for immunotherapy. Thus, intervention studies geared to manipulate key metabolic checkpoints during inflammatory immune responses have garnered widespread interest, particularly in the context of vaccine development and for therapeutic purposes.

## Metabolic Requirements of T Cells

The synthesis of lactate is essential to allow T cells to have elevated glycolytic flux. If one considers glucose as a key source of carbon for cellular biosynthesis, the export of large amounts of glucose-derived carbons as lactate seems wasteful and inefficient; however, rapidly proliferating T cells prefer to switch their metabolism to fermenting glucose to lactate, even in the presence of oxygen (7). As indicated above, while the increased glucose uptake is used to fuel other biosynthetic demands, this only accounts for a small fraction of the high amount of acquired glucose (11, 12). Instead, new evidence suggests that the surplus glucose can flux through the HBP that yields UDP-GlcNAc (13). UDP-GlcNAc is a substrate for *O*-GlcNAc transferase (OGT) that functions to add *O*-linked-β-*N*-acetylglucosamine (*O*-GlcNAc) to the serine/threonine residues of intracellular proteins. Interestingly, *O*-GlcNacylation was shown to stabilize the expression of the proto oncogene, c-myc in T cells. Deletion of OGT led to a failure in c-myc stabilization and significantly impaired T cell clonal expansion and self-renewal (13). This highlighted the critical role that glucose and glutamine funneled through HBP has on T cell biology.

One key outcome of the T cell response is formation of immunological memory in the form of memory T (Tm) cells. Tm cells are long-lived cells that primarily use oxidative metabolism: they have low rates of aerobic glycolysis and rely on mitochondrial oxidative phosphorylation (OXPHOS) (1). Tm cells have a higher mitochondrial mass than Tn cells, providing them with greater respiratory capacity that allows them to engage high rates of OXPHOS to generate ATP immediately following restimulation (15). However, OXPHOS is fueled differently in Tm cells than Tn cells. Tm cells adopt a metabolic configuration in which glucose is used to fuel fatty-acid synthesis, and these fatty acids are stored as triglycerides before being metabolized *via* β-oxidation to fuel OXPHOS (15–17). From a bioenergetics standpoint, this would seem like an inefficient mechanism to fuel OXPHOS as fatty-acid synthesis uses ATP. Nonetheless, this seemingly futile cycle of fatty-acid synthesis and fatty-acid oxidation (FAO) is important for Tm survival (16, 17). One advantage is that it allows Tm to concurrently engage glycolysis and OXPHOS, thus maintaining the machinery for rapid induction of metabolic flux through these pathways upon antigen recognition, and so facilitating rapid functional responses that are characteristic of Tm (15, 18).

Glucose and lipid metabolism are important in determining CD8^+^ T cell effector (Teff) versus Tm differentiation. Pharmacological inhibition of glycolysis promotes Tm formation, and mice deficient in lipid metabolism fail to generate Tm (19). Additionally, modulating the signaling pathways that control glucose and lipid metabolism, such as mTORC1 and AMPK signaling, affects the balance of CD8^+^ Teff and Tm formation (20, 21).

Cellular metabolism directly regulates T cell fates and functions as metabolic enzymes and metabolites regulate T cell signaling and effector molecule production. For instance, the glycolytic enzyme glyceraldehyde 3-phosphate dehydrogenase (GAPDH) binds to the three prime untranslated region (3′UTR) of interferon-γ (IFN-γ) and IL-2 mRNA and regulates their expression (22). This function is likely not unique to GAPDH, as numerous metabolic enzymes from diverse metabolic pathways, including fatty-acid synthesis, are mRNA binding proteins (23). Additionally, the glycolytic metabolite phosphoenolpyruvate is an important signaling molecule in T cells that controls the duration of T cell receptor (TCR)-induced Ca^2+^ signaling (24).

## Differential Use of Glucose or Fatty-Acid Metabolism Regulates the Development and Optimal Functions of CD8^+^ Teff and Tm Cells, Respectively

**Figure 1:**
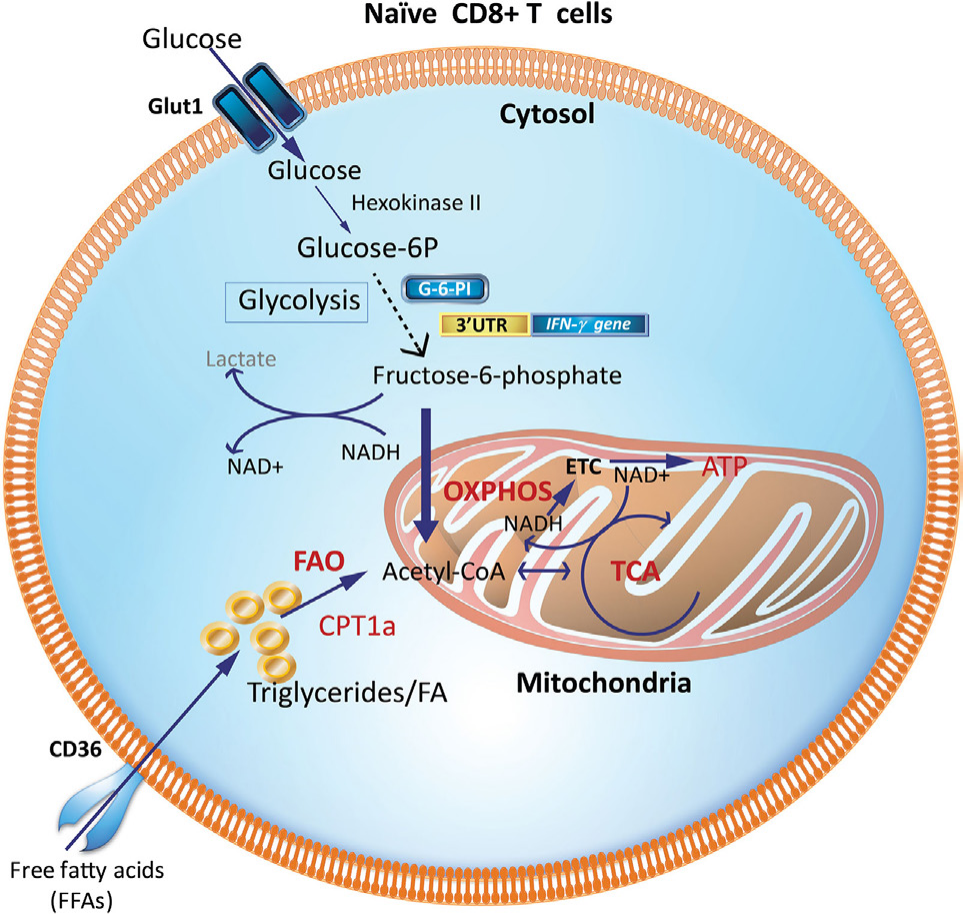
Metabolic requirements of naïve CD8^+^ T cells (Tn). Glucose and fatty acids are major nutrient sources for immune cells and are metabolized predominantly via OXPHOS and FAO in naïve T cells (Tn). Glycolytic enzymes like GAPDH when not involved in glycolysis may engage the 3′-UTR of effector cytokines like IFN-γ and IL-2 and suppress their production. CPT1a, carnitine palmitoyltransferase 1A; FAs, fatty acids; FAO, fatty-acid oxidation; GAPDH, glyceraldehyde 3-phosphate dehydrogenase; G-6-PI, glucose-6-phosphate isomerase; IFN-γ, interferon-γ; OXPHOS, oxidative phosphorylation; TCA, tricarboxylic acid cycle; 3′UTR, three prime untranslated region.

During the resting phase, CD8^+^ Tn cells primarily use OXPHOS driven by FAO (Figure 1). While glycolysis only generates two molecules of ATP, OXPHOS generates more ATPs to maintain homeostasis (8, 10, 25, 26). This is consistent with their high expression of the hallmark enzyme of FAO, carnitine palmitoyl transferase 1a (Cpt1a) and low expression of the first-step glycolytic enzyme, hexokinase-2 (27). However, upon encountering antigen, Teff development requires a reprogramming to glycolysis that is accompanied by, and supports, rapid proliferation (clonal expansion) and acquisition of specialized effector functions (cytokine production and cytotoxicity) (16, 28). The ability to access key nutrients during the effector phase depends on the expression of dedicated transporters. The high-affinity glucose transporter 1 (Glut-1) is the major glucose transporter in T cells (29, 30) and Glut-1 protein expression is upregulated during TCR engagement and Teff generation (3, 27, 31).

**Figure 2:**
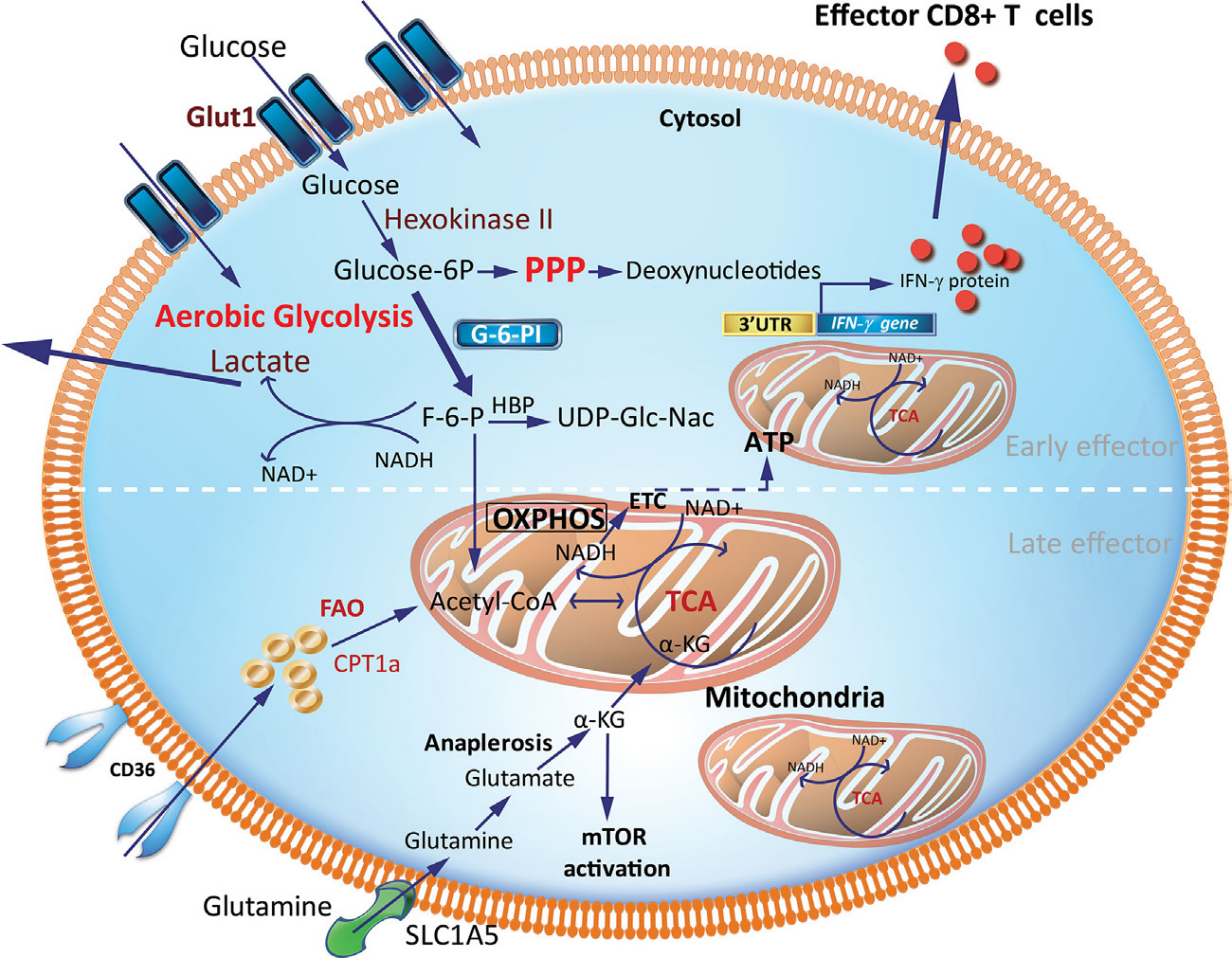
Metabolic requirements of effector CD8^+^ T cells (Teff). Early generation of effector cells requires a metabolic reprogramming from OXPHOS and FAO toward aerobic glycolysis with increased OXPHOS driven by FAO in late effector stage. A hallmark of aerobic glycolysis is elevated aerobic glycolysis and secretion of lactate. PPP intermediates are elevated to support synthesis of nucleotides, proteins and lipids for cell growth, proliferation, and effector responses. In activated effector cells, the HBP may utilize F-6-P to form glucosamine-6-phosphate by glutamine-fructose-6-phosphate transaminase in the presence of glutamine. This is a key step in the production of UDP-GlcNAc, a substrate for protein glycosylation. GAPDH a key glycolytic enzyme may disengage the 3′-UTR of effector cytokines to perform its metabolic role, thereby inducing cytokine production. Activated effector cells also increase glutamine uptake which generates α-KG via anaplerosis. This α-KG supports the TCA cycle under condition of limited pyruvate and acetyl-CoA. α-KG may also directly activate mTOR. CPT1a, carnitine palmitoyltransferase 1A; FAs, fatty acids; FAO, fatty-acid oxidation; F-6-P, fructose-6-phosphate; GAPDH, glyceraldehyde 3-phosphate dehydrogenase; HBP, hexosamine biosynthetic pathway; α-KG, α-ketoglutarate; OXPHOS, oxidative phosphorylation; PPP, pentose phosphate pathway; mTOR, mechanistic target of rapamycin; TCA, tricarboxylic acid cycle; UDP-GlcNAc, uridine diphosphate *N*-acetyl glucosamine; 3′-UTR, three prime untranslated region.

Teff generated early during an immune response are more efficient at glucose uptake, as measured by 2-NBDG, a fluorescent glucose analog and Seahorse technology (Figure 2). The Seahorse Extracellular Flux Analyzer can be used to profile the metabolic phenotypes of cells by simultaneously measuring the extracellular acidification rate (ECAR) and oxygen consumption rate (OCR) in real time from a relatively low number of cells. ECAR and OCR are proportional to aerobic glycolysis and OXPHOS, respectively. In particular, ECAR quantifies proton concentration sensed by electrode probes as a consequence of lactate production and is therefore used as a proxy for aerobic glycolysis. Using Seahorse, it was shown that CD8^+^ Teff, generated by activation of CD8^+^ T cells in the presence of IL-2, have almost fourfold greater glycolytic capacity as indicated by increased ECAR than cells activated in the presence of IL-15, which resemble Tm (32). This is also commensurate with the observation that murine CD8^+^ Teff generated after infection with *Listeria monocytogenes* has a higher glycolytic capacity than Tn and Tm cells. In addition, Teff have a high expression of key enzymes in the glycolytic pathway, including phosphoglycerate mutase and lactate dehydrogenase. CD8^+^ Teff also have significantly higher intracellular levels of glycolytic metabolites, such as glucose-6-phosphate and phosphoenolpyruvate, than CD8^+^ Tn (27). Notably, inhibiting glycolysis in CD8^+^ Teff with the glucose analog 2-deoxyglucose (2-DG), an inhibitor of hexokinase-2, suppresses IFN-γ, tumor necrosis factor (TNF)-α, and granulocyte and macrophage-colony stimulating factor (GM-CSF) cytokine production in response to anti-CD3 and anti-CD28 TCR stimulation (28, 33). 2-deoxyglucose also abrogated the cytolytic machinery (i.e., the expression of perforin and granzymes B and C), which translated to impaired cytotoxicity of CD8^+^ Teff against P815-B7.1 tumor cells (28). Development of early CD8^+^ Teff requires a metabolic switch from OXPHOS in CD8^+^ Tn to glycolysis to sustain Teff homeostasis and function (Figure 2). In contrast, when an infection is cleared, late CD8^+^ Teff become less reliant on glycolysis and are more efficient at uptake of long fatty-acid chains such as palmitate that drives FAO (16). This is consistent with the high expression of CD36, which binds long-chain fatty acids and low-density lipoprotein (LDL) (16). In sum, the efficient generation of effector cells demands a switch from FAO to glycolysis to generate early Teff and, thereafter, a switch to OXPHOS driven by FAO for late Teff (Figures 2 and 4).

## Mitochondrial OXPHOS Fueled by FAO Enhancing CD8^+^ Tm Development and Recall Capacity

**Figure 3:**
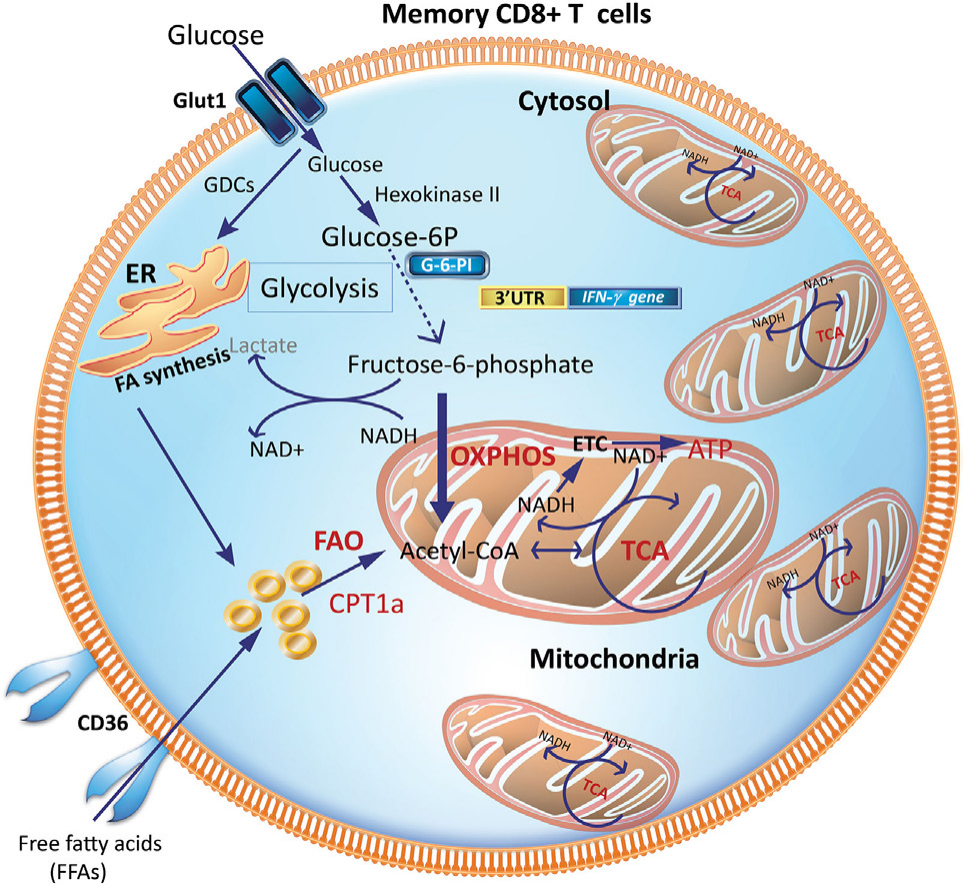
Metabolic requirements of memory CD8^+^ T cells (Tm). Increased mitochondrial density and a metabolic configuration toward OXPHOS and FAO allows Tm cells to concurrently engage in both glycolysis and OXPHOS for rapid metabolic flux upon antigen recognition to facilitate a rapid and robust functional response. GDCs are used for FA synthesis in the ER, which can undergo FAO to generate acetyl-CoA to fuel the TCA cycle to generate ATP. CPT1a, carnitine palmitoyltransferase 1A; ER, endoplasmic reticulum; FAs, fatty acids; FAO, fatty-acid oxidation; GDCs, glucose-derived carbons; OXPHOS, oxidative phosphorylation; TCA, tricarboxylic acid cycle.

**Figure 4:**
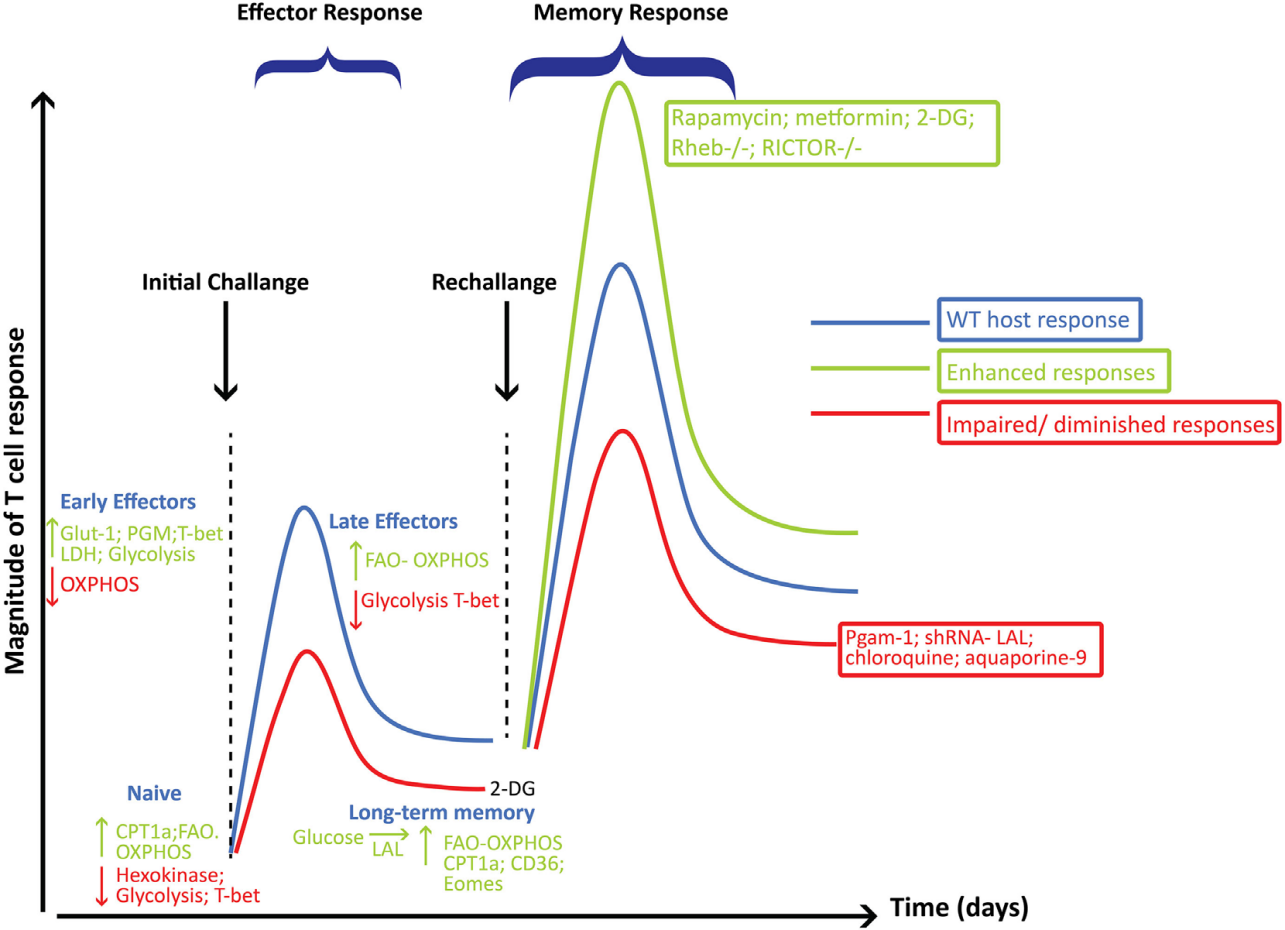
Metabolic reprogramming playing a critical role in governing the generation and function of CD8^+^ effector (Teff) and memory cells (Tm). This figure summarizes some of the major pathways and molecular players involved at each stage of CD8^+^ T cell differentiation following viral infections and how these pathways influence the magnitude of the Teff and Tm responses. Metabolic pathways, key enzymes, transcription factors, or intervention strategies that are decreased or that would hamper the development of Tm are shown in red, while those that enhance Tm responses are shown in green.

Tm cells acquire a metabolic phenotype that depends on FAO and OXPHOS (Figures 3 and 4). Overexpressing the glycolytic enzyme phosphoglycerate mutase 1 (*pgam1*) in antigen-specific CD8^+^ T cells impaired their expansion in response to vaccinia virus upon rechallenge (27). In stark contrast, CD8^+^ T cells treated with 2-DG, to suppress glycolysis during T cell priming, retained a memory phenotype and had an augmented production of IFN-γ and TNF-α and, more importantly, were more efficient at controlling tumor development (27). This suggested that an “always on” glycolytic switch impedes functional development of CD8^+^ Tm (27). Also, adoptive transfer of 2-NBDG^lo^CD8^+^ T cells had a greater capacity for long-term survival than 2-NBDG^hi^ cells. Thus, higher uptake of glucose is associated with short-lived effector cells (SLECs), whereas CD8^+^ T cells that utilize less glucose are endowed with the capacity to become long-lived memory cells (Figure 4).

These conclusions contrast with a recent report showing that a constitutively active glycolytic pathway did not impair the differentiation and function of Tm CD8^+^ T cells, but that augmented glycolysis provided sufficient energy to drive Tm development (34). This study used a mouse model with a genetic deletion of the von-Hippel Lindau (VHL) protein. Deletion of VHL, which negatively regulates hypoxia-inducible factor (HIF), leads to constitutive HIF activity and high HIF activity in turn leads to significantly augmented glycolysis accompanied by reduced OXPHOS. This study provided a novel approach to deciphering whether metabolic preference was central to memory cell generation and function. It showed that selectively enhancing glycolysis and diminishing OXPHOS did not hamper the development or function of Tm cells, but importantly, wild-type and VHL-deficient cells had similar levels of ATP. One explanation that the authors provide to reconcile the observations from this study with those showing that fatty-acid-fueled OXPHOS is essential for Tm development is that the availability of sufficient levels of ATP may play a key role in regulating cell fate decisions. In this regard, CD8^+^ Teff destined to differentiate into Tm must meet a certain “ATP threshold” to enable their differentiation and optimal function as Tm irrespective of the metabolic pathway utilized to generate the required levels of ATP. Therefore, it can be reasoned that without genetic or pharmacologic manipulation, late Teff may specifically undergo metabolic reprogramming in favor of FAO because this metabolic pathway selectively yields higher levels of ATP. In the case of genetic manipulation such as VHL deletion, constitutively active glycolysis may meet the required ATP threshold that is sufficient to drive normal Tm differentiation (34). It remains to be determined whether VHL deficiency in mice may also have other indirect effects on metabolic reprogramming.

Interestingly, CD8^+^ Tm cells acquire extracellular glucose and channel it to synthesize fatty acids in the endoplasmic reticulum (ER), a process dependent on the enzyme, lysosomal acid lipase (LAL). LAL is critical in hydrolyzing cholesteryl esters and triglycerides within LDL particles into free cholesterol and free fatty acids (FFAs) (35, 36). The newly synthesized fatty acids from glucose-derived carbon are subsequently utilized for mitochondrial FAO and OXPHOS (Figure 3). Inhibiting FAO, by transduction of transgenic CD8^+^ T cells with retrovirus expressing short hairpin RNA (shRNA) against LAL, markedly impaired the development of CD8^+^ Tm cells (16). Similarly, inhibiting LAL with shRNA or treatment with chloroquine or genetic deletion of the glycerol channel, aquaporin 9 in mice, compromised the survival of CD8^+^ Tm generated *in vitro* and *in vivo* (16, 17, 37). Similarly, exogenous uptake of FFAs by the lipid chaperone proteins, FABP4 and FABP5, is critical for the long-term maintenance and survival of long-lived tissue-resident memory (TRM) cells in the skin where there is a ready source of lipids. Notably, antigen-specific CD8^+^ T cells lacking expression of FABP4 and FABP5 lost their functional competence in conferring protection against skin vaccinia virus infection (38). Pharmacologic inhibition of FAO by etomoxir, which inhibits Cpt1a, similarly impaired CD8^+^ T cell-mediated protection against vaccinia virus infection, suggesting that acquisition of FFAs that subsequently fuel FAO-driven OXPHOS is closely coupled to the long-term survival and function of TRM cells (38).

The switch from glycolysis to OXPHOS may also be promoted by the essential amino acid, L-arginine (39). *In vitro* TCR-activated human CD4^+^ T cells rapidly acquire and consume L-arginine, which leads to an increase in molecular intermediates of the urea cycle and nucleotides, sugar derivatives, and other amino acids. L-arginine uptake inhibited expression of Glut-1 and enzymes essential in the glycolytic pathway (39). Consequently, glucose uptake and glycolysis were diminished, but this was accompanied by an increase in the tricarboxylic acid cycle (TCA) metabolites and mitochondrial OXPHOS. Pre-treatment of OT-I T cells with L-arginine permitted more robust *in vivo* responses against B16-OVA tumor cells, and in general, L-arginine promoted a central memory-like phenotype that may be, in part, due to an increase in OXPHOS accompanied by the decrease in glycolysis (39). These data suggest that limiting glycolysis and promoting OXPHOS through FAO during the contraction of Teff and transition to memory pave the way for optimal development and function of CD8^+^ Tm.

A recent study interrogating why Tm cells primarily rely on FAO while Teff use aerobic glycolysis showed that mitochondrial remodeling plays a critical role in instructing T cell metabolic reprogramming (40). Mitochondrial ultrastructural analysis using electron microscopy revealed that unlike Teff cells which had punctate mitochondria that undergo fission, Tm cells had densely packed fused mitochondria which was commensurate with a high-protein expression of the mitochondrial inner membrane fusion protein, optic atrophy 1 (Opa1) (40). Opa1 was shown to be critical for promoting mitochondrial fusion and cristae remodeling in Tm cells as its absence led to disrupted cristae organization and reduced electron transport chain activity. This lead to the inability to generate Tm cells through inefficient FAO, reduced OCR/ECAR ratios, and reduced spare respiratory capacity (SRC), a parameter that quantifies a cell’s ability to respond to increased energy demand and depends on FAO in CD8^+^ Tm (40). While absence of Opa1 did not alter Teff cell generation, enforcing mitochondrial fusion in Teff cells either pharmacologically using the fusion promoter, hydrazone M1 and the fission inhibitor, Mdivi-1, or through genetic overexpression of Opa1, led to enhanced OXPHOS and SRC, and promoted the development of Tm cells even under Teff cell differentiation conditions. However, Opa1-deficient Teff were shown to utilize FAO at a similar extent as controls, suggesting that Opa1 is dispensable for FAO. As fine-tuning metabolism has enormous implications for immunotherapy, pharmacologically enforcing mitochondrial fusion in OT-I Teff significantly enhanced their ability to confer better protection in mice harboring EL4-OVA expressing tumors compared with recipients of control treated cells. Taken together, this suggests that mitochondrial dynamics through fission and fusion is a key player in the metabolic reprogramming promoting Teff versus Tm cells, respectively (40).

Critically, while a metabolic switch to FAO may be necessary for development of Tm cells, it does not translate to a complete shutdown of the glycolytic pathway. In fact, effector memory CD8^+^ T cells, which are considered to be the most efficient at executing recall responses, depend on glycolysis for early production of IFN-γ (18). This finding does not contradict other reports showing that FAO is critical for Tm function, but highlights an important dependence of CD8^+^ T cells on glycolytic flux to support their effector function and to enable fatty-acid synthesis for FAO (Figure 4).

## mTOR, the Serine–Threonine Kinase That Integrates Various Metabolic and Inflammatory Cues, is a Central Player in CD8^+^ Teff and Tm Development

The evolutionarily conserved serine/threonine protein kinase, mechanistic target of rapamycin (mTOR), integrates various stimuli to activate the metabolic pathways that ultimately drive cellular growth and effector functions. mTOR consists of two functionally distinct multiprotein complexes, mTORC1 and mTORC2. RAPTOR (regulatory-associated protein of mTOR) and RICTOR (rapamycin-insensitive companion of mTOR) are the essential core adaptor components of mTORC1 and mTORC2, respectively (41). Mechanistic target of rapamycin complex 1 (mTORC1) responds to amino acids, oxygen, energy, and growth factors and is acutely sensitive to the inhibitor, rapamycin (41). On the other hand, mTORC2 responds to growth factors and modulates cellular survival and cytoskeletal restructuring and is insensitive to acute rapamycin treatment, although chronic exposure can disrupt its molecular structure (41). The role of mTOR as a key checkpoint in metabolic reprogramming warrants investigating its role in CD8^+^ Teff and Tm development. Indeed, a large body of evidence supports the view that mTOR is a central player in regulating CD8^+^ Teff and Tm generation by controlling metabolism and the key T-box transcription factors, T-bet, and Eomesodermin (Eomes) that promote Teff and long-term memory, respectively (42–47).

Inhibiting mTORC1 activity with rapamycin or the AMPK activator metformin augmented the development of memory precursor effector cells (MPECs) and long-lived Tm cells that were more functionally robust at eliciting protective immunity during recall responses (19, 20). Moreover, asymmetric partitioning of mTORC1 during cellular division of CD8^+^ T cells leads to the generation of two daughter cells, CD8^hi^ and CD8^lo^, cells with different mTORC1 activities and metabolic requirements (48). CD8^hi^ cells with higher mTORC1 activity have a higher glycolytic dependency and develop into effector cells while being inefficient at generating long-term Tm cells. In contrast, CD8^lo^ cells with significantly lower mTORC1 activity are less glycolytic, have a higher mitochondrial mass, and develop more superior long-lived Tm cells (48, 49). Complementing these findings, T cells deficient in tuberous sclerosis complex 1 or 2 (TSC1/2), which inhibits mTORC1 activity, are highly glycolytic and failed to develop long-term antigen-specific Tm, but were more proficient in the development of effector cells that were largely KLRG1^+^ cells, also known as SLECs (49, 50). However, treating transferred OT-I TSC2^−/−^ cells with rapamycin *in vivo* was associated with a higher expression of CD127 and the transcription factor Eomes, which is critical in development of Tm, and more importantly, rapamycin treatment enhanced their recall response (Figure 4).

In keeping with the prominent role of mTORC1 in regulating metabolism, TSC2-deficient CD8^+^ T cells that have high levels of mTORC1 activity are highly glycolytic, which is enabled by the high-level expression of Glut-1 and phosphofructokinase. In contrast, CD8^+^ T cells deficient in Rheb, an mTORC1 activator, had a decreased glycolytic capacity. This biological phenotype can be relegated to the capacity of mTORC1 to control glycolysis through the hypoxia-inducible factor-1 (HIF-1) transcription factor complex, which in turn regulates the expression of the key glycolytic enzymes and glucose transporters (3, 51). Commensurate with reduced glycolysis, Rheb-deficient CD8^+^ T cells had higher mitochondrial mass and SRC (3, 15, 32). Notably, greater mitochondrial mass enforces efficient SRC and permits a more robust recall response (15, 32).

Until recently, little was known about how mTORC2 influences CD8^+^ Teff and Tm generation. RICTOR-deficient T cells have increased expression of CD127 and Eomes and lower expression of T-bet, suggesting that mTORC2 constrains development of MPECs (49, 52). In addition, RICTOR deficiency augmented the development of long-term Tm cells, which were superior in cytokine production (49). As anticipated, enhanced development of Tm was closely related to a higher expression of Cpt1a and SRC. Thus, mTOR activity is central to regulating the differentiation of CD8^+^ Teff and Tm cells, by acting as a rheostat for fine-tuning metabolic reprogramming (19, 46, 49).

In terms of metabolic reprogramming, in the absence of either mTORC1 (Rheb^−/−^) or mTORC2 (Rictor^−/−^) activity, CD8^+^ T cells demonstrate enhanced mitochondrial mass, OCR, SRC, and high expression levels of Cpt1a, all of which promote the optimal development and function of Tm with an oxidative phenotype (49, 52). Notably, in the absence of mTORC2, CD8^+^ T cells have a marked increase in glycolytic flux (ECAR) in memory CD8^+^ T cells (49). In contrast, loss of mTORC1 leads to a significant decrease in ECAR, Glut-1, and phosphofructokinase (49). Taken together, the loss of mTORC2 orchestrates a metabolic profile that supports the development of both Teff and Tm responses. Therefore, it appears that mTORC1 rather than mTORC2 is dispensable for metabolic programming of CD8^+^ T cells toward a glycolytic phenotype.

Our current knowledge on T cell metabolism has been largely restricted to acute infections in mouse models. Knowledge on which metabolic pathways are critical during chronic infections in humans is limited but has recently gained momentum particularly in the hepatitis B and HIV fields.

## Metabolic Reprogramming of CD8^+^ T Cells During Chronic HBV and HIV Infection

According to the World Health Organization (WHO), approximately 240 million people in the world are infected with hepatitis B virus (HBV). Chronic infection with HBV leads to serious liver disease, and infected individuals are at a high risk of liver cirrhosis, hepatic cellular carcinoma, and liver failure. Current treatments that suppress viral replication must be continued on a life-long basis, and thus, there is a significant need to define a therapy that will cure or control HBV infection. In addition to targeting the virus directly, the immune system can be targeted therapeutically. The immune system during chronic viral infections becomes impaired or “exhausted” (53). It becomes overwhelmed with continuous high-level antigen exposure and associated inflammation, so that immune cells are no longer functionally effective.

This is particularly true for HBV-specific CD8^+^ T cells (54). The metabolism of CD8^+^ T cells is affected during chronic HBV infection and likely contributes to their impaired functions. HBV-specific CD8^+^ T cells were unable to efficiently metabolize glucose by OXPHOS and had associated mitochondrial dysfunction, including a lower mitochondrial membrane potential and impaired OXPHOS capability. However, IL-12 reversed this and boosted OXPHOS in HBV-specific CD8^+^ T cells (55). Interestingly, genes encoding key components of the mitochondrial electron transport chain that drive OXPHOS were lower in exhausted human CD8^+^ T cells with chronic HBV infection than in patients who controlled their infection (56). HBV-specific CD8^+^ T cells from these patients also had less mitochondrial polarization but higher mitochondrial reactive oxygen species (ROS) than resolved patients or healthy controls. Notably, using antioxidants to reverse mitochondrial dysfunction in exhausted HBV-specific CD8^+^ T cells increased the expression of mitochondrial electron transport chain components and enhanced their IFN-γ and TNF-α producing capacity after *in vitro* HBV-core-peptide stimulation (56). These studies suggest that enhancing mitochondrial function reverses the impaired functionality associated with exhaustion in human CD8^+^ T cells (56).

Nearly 40 million people worldwide are living with HIV. Of these only 17 million have access to antiretroviral treatment (ART) to suppress viral load and halt disease progression to AIDS (57). The critical role of CD8^+^ T cells in controlling HIV replication has been noted in simian immunodeficiency virus (SIV) infection where depletion of CD8^+^ T cells exacerbates viral loads in infected rhesus monkeys (58). Similarly, in HIV-infected patients, escape mutations in MHC-I restricted epitopes reflect a key role that HIV-specific CD8^+^ T cells have in controlling viral loads by imposing a selection pressure (59). HIV-specific CD8^+^ T cells in acute infection fail to generate biosynthetic and energy requirements, leading to augmented apoptosis (60). In particular, the reduced survival during acute HIV infection was attributed to increased mitochondrial dysfunction and significant down-regulation of the NRF2-mediated oxidative stress response, which degrades oxidized proteins. Compared with chronically activated HIV-specific CD8^+^ T cells, in acute HIV infection, CD8^+^ T cells have less mTOR activity, but more protein ubiquitination and purine metabolism (60). Additionally, reduced expression of CD127 led to reduced STAT5 phosphorylation after IL-7 stimulation *in vitro*. This was associated with reduced downstream activation of PI3K, AKT, and Bcl-2 expression after IL-7 stimulation and reduced cytokine production (IFN-γ, TNF-α, and IL-2) after cognate peptide stimulation (60). Although not examined comprehensively in the context of glucose or lipid metabolism, the work highlighted here argues for a link between metabolic and transcriptional defects and impaired CD8^+^ T cell functions during acute HIV infection.

With respect to chronic HIV infection, evidence of metabolic reprogramming in CD8^+^ T cells has been lacking. However, chronic antigen stimulation is accompanied by upregulation of negative regulatory checkpoints, including PD-1 and CTLA-4 (61). Specifically, in a model of chronic TCR stimulation in CD4^+^ T cells, ligation of PD-1 augmented Cpt1a expression, SRC, and FAO, but inhibited glycolysis and glutaminolysis where glutamine is deaminated to produce glutamate which is then converted to α-ketoglutarate (α-KG). α-KG is critical for fueling the TCA cycle and for maintaining ATP production. Furthermore, increased glutamine uptake during glycolysis is essential as an amine donor to generate glucosamine-6-phosphate from fructose-6-phosphate by the rate limiting HBP enzyme glutamine-fructose-6-phosphate transaminase (62). This suggests that the HBP can compete with glycolysis for fructose-6-phosphate and with glutaminolysis for glutamine in activated T cells. CTLA-4 engagement also inhibited Glut-1 and glutamine transporters, leading to reduced glycolysis and glutaminolysis. Implicit in these findings is that PD-1 and CTLA-4 ligation may prevent robust generation of effector cells by inhibiting glycolysis and glutaminolysis, which are critical for supporting their differentiation. Given this and the previous discussion of CD8^+^ T cell metabolism in HBV, chronic infections, such as HBV and HIV, lead to altered glycolysis, OXPHOS, and increased mitochondrial dysfunction through the high-level expression of ROS. Future studies will need to address this essential and unresolved question of how HIV influences the metabolism of CD8^+^ Teff and Tm in patients with or without ART.

Understanding how cellular metabolism links to dysfunctional immune responses in acute and chronic diseases will help to tailor therapies with maximum beneficial effects through the rejuvenation of T cell functions.

## Metabolic Reprogramming in CD4^+^ T Cells During HIV Infection

CD4^+^ T helper (Th) cell lineage differentiation is closely coupled to specific metabolic pathways. For example, glycolysis is critical for supporting Th1, Th17, and Th2 differentiation, whereas FAO enhances regulatory T cell (Treg) differentiation (14, 63–66). Interestingly, new data suggest that aerobic glycolysis and glutaminolysis have critical roles in driving the differentiation of Th cells. In activated CD4^+^ T cells, aerobic glycolysis occurs in parallel with glutaminolysis where glutamine is converted to glutamate. It has been shown that in T cells, aerobic glycolysis, and glutaminolysis promote pro-inflammatory Th17 cell fate at the expense of iTreg development. As glucose and glutamine drive the HBP, aerobic glycolysis and glutaminolysis effectively starve and lower the availability of the precursors required to drive the HBP that produces UDP-GlcNAc. This leads to reduced *N*-glycan branching in T cells and in turn promotes Th17 development over inducible Treg (iTreg) differentiation (62). Specifically, supplementing T cells with GlcNAC blocked Th17 differentiation and promoted the differentiation of iTregs even in the presence of Th17 polarizing conditions by blocking the endocytic loss of CD25 (62). To demonstrate the utility of GlcNAC in regulating Th17 cell differentiation, oral delivery of GlcNAc in a mouse model of experimental autoimmune encephalomyelitis (EAE) where pathogenesis is dependent on Th17 responses significantly impeded disease progression. This provides evidence to show that manipulating metabolic pathways can rheostatically fine tune T cell fates and function (67). On another front, glutamine starvation was shown to drive the differentiation of iTreg cells at the expense of Th1 cells, which is a consequence of reduced α-KG (68). This observation also underscores the critical role of TCA intermediates like α-KG to regulate the epigenetic status of activated T cells. Indeed, increased glutaminolysis is essential for tet2-mediated demethylation and regulation of the epigenetic landscape required for Th1 differentiation (68). It is important to keep in mind that lactate, a byproduct of aerobic glycolysis is commonly used as a major readout of glycolysis. However, since glutamine can be metabolized to lactate, the level of glycolytic metabolism may be overestimated in many of the currently used protocols. Thus, total lactate production may be a cumulative measure of both glycolysis and glutaminolysis.

How metabolic pathways regulate CD4^+^ T cell differentiation has been described in great detail in a number of excellent reviews (14, 63–66). In the following section, we will focus on how CD4^+^ T cell metabolism is altered by HIV infection.

## Immune Activation and Exhaustion are Key Hallmarks of HIV Infection

The exact mechanisms by which HIV evades the immune system, causing immune deterioration, persistent inflammation, and reservoir establishment, have eluded HIV researchers for over three decades. These unresolved questions are major obstacles for the development of effective preventative vaccines, therapeutics to induce robust immunological recovery, and a cure for HIV. Immunometabolism has challenged old paradigms and fostered novel hypotheses and dynamic insights into HIV pathogenesis.

CD4^+^ T cells are preferentially targeted by HIV (69), although subsets of CD16^+^ monocytes and macrophages may also be infected (70, 71). In acute HIV infection, CD4^+^ T cells rapidly decline in the gut and in the periphery, followed by partial CD4^+^ T cell recovery, and then an inexorable depletion of this cell population over time in untreated individuals, leading to AIDS within 6–10 years (72, 73), though this is not universal. Acute HIV infection is associated with high levels of CD4^+^ and CD8^+^ T cell activation and exhaustion, which is often maintained throughout chronic infection due to prolonged exposure to antigenic and inflammatory signals even in anti-retroviral-treated individuals (74, 75). Markers of T cell activation, such as CD38 and HLADR, remain elevated above normal in HIV positive individuals with durable viral suppression on ART (76). Moreover, elevated expression of co-inhibitory receptors, such as PD-1, TIGIT, CD160, and 2B4, is associated with functionally exhausted HIV-specific and non-specific CD8^+^ T cells with reduced proliferative capacity and impaired effector functions (77–80).

## Impact of CD4^+^ T Cell Metabolism on HIV Disease Progression

CD4^+^ T cells from HIV positive individuals exhibit a glycolytic phenotype, featuring significantly increased cell-surface expression of Glut-1, abnormally high hexokinase activity, and lactate production (81). This metabolic state differs from quiescent cells where OXPHOS predominates as the major pathway to provide energy and is reminiscent of the metabolic profile seen in tumor cells. This may be interpreted as a metabolic sequelae to sustain activation, proliferation, and effector cytokine production, such as IFN-γ (82). Indeed, high Glut-1 expression on CD4^+^ T cells is positively associated with markers of T cell activation (e.g., CD38, HLA-DR), and low CD4^+^ T cell counts in untreated patients (81). Similarly, individuals categorized as immunological non-responders (i.e., treated HIV positive persons with low CD4^+^ T cell counts despite prolonged HIV suppression) have a high frequency of circulating CD4^+^Glut-1^+^ T cells (81). Interestingly, a low frequency of circulating CD4^+^Glut-1^+^ T cells was seen in HIV long-term non-progressors, who maintain normal CD4^+^ T cell counts despite being infected with HIV for more than 10 years without ART (81). Evidence shows that Glut-1 is selectively required for CD4^+^ T cell activation (83) and the Glut-1-mediated metabolic pathway is a critical regulator of HIV infection in CD4^+^ T cells (81, 84). As activated CD4^+^ T cells are the favored targets of HIV, one could speculate that Glut-1 is an intrinsic marker of an activated state essential for HIV infection. *In vivo* Glut-1 expression on CD4^+^ T cells in HIV positive individuals is regulated, at least in part, by cytokines, such as IL-7, which is also implicated in Glut-1 cell-surface trafficking *via* STAT5-mediated Akt activation (30).

## Regulators of Glucose Metabolism in CD4^+^ T Cells During HIV Infection

Metabolic exhaustion owing to the SRC of CD4^+^ T cells and directed by hyperactivation of PI3K-Akt-mTOR axis might contribute to CD4^+^ cell loss in HIV infection (14). Indeed, glycolytic flux is increased in *in vitro*-HIV-infected primary CD4^+^ T cells (85). Furthermore, infected primary CD4^+^ T cells cultured in galactose had a survival advantage over those cultured in glucose, and this was associated with reduced caspase 3 activity and apoptosis. Interestingly, glycolytic flux proceeded without alteration in levels of key glycolytic enzymes, such as hexokinase 2, GAPDH, and pyruvate kinase M2 (PKM2), leaving the authors to speculate that glycolysis may ensue by way of allosteric regulation or post-translational modification of glycolytic enzymes. PPARy-dependent fatty-acid uptake critically regulated metabolic reprogramming in TCR triggered CD4^+^ T cells (86), but the involvement of this pathway in T cell metabolic activation in HIV infection is unknown.

## Effects of mTOR on HIV Infectivity and Persistence

Mechanistic target of rapamycin complex 1 regulates CCR5 expression required for viral entry of CCR5 (R5)-tropic HIV, and mTORC2 controls phosphorylation of protein kinase C for nuclear Factor-κB (NF-κb) induction of HIV transcription (87). Dual targeting of mTORC1/2 complex with INK128 inhibited replication of R5-tropic and CXCR4 (X4)-tropic HIV strains BaL and HXB2, respectively, in primary peripheral blood lymphocytes (PBLs). However, INK128 activity was more potent against R5 than X4 strains due to reduced CCR5 levels, which interrupt fusion of CD4^+^ T cells with the R5 envelope (87). Regulation of mTORC2 by INK128 interferes with NF-κB induction that may prevent recruitment of the transcription factor positive transcription elongation factor b to the HIV long-terminal repeat and suppress viral transcription. Remarkably, INK128 inhibited a multidrug-resistant HIV clone NL4329129-2, which carries the reverse transcriptase gene amplified from a patient with multidrug-resistant HIV. More importantly, INK128 suppressed HIV in a preclinical animal model of NOD/SCID/IL-2Rγ^null^ mice reconstituted with human PBLs and infected with BaL (87). Thus, mTOR inhibitors in cancer therapy may be considered for repurposing to help control HIV in patients who have failed ART therapy due to drug resistance.

Another critical hallmark of HIV is establishment of a stable latently infected HIV reservoir within days of infection, predominantly within the memory CD4^+^ T cell compartment (88). The role of immunometabolism in HIV latency was highlighted in which a genome-wide screen on different HIV latency models and HIV-infected patient cells uncovered an essential role of mTOR complex in HIV latency. mTOR inhibitors Torin1 and pp242 targeting mTORC1 and mTORC2 strongly suppressed latent HIV reactivation after TCR-mediated CD4^+^ T cell activation. Inhibiting these complexes was associated with suppressed CDK9 phosphorylation and activation that in turn abrogated Tat-dependent and -independent transactivation of the HIV promotor (87). This corroborated a recent study showing that the HIV regulatory gene, tat, hyperactivates mTORC1 activity in a PI3K-dependent manner for synthesis of biomolecules for virion production and latent viral reactivation. This is mediated *via* activation of carbamoyl-phosphate synthetase 2, aspartate transcarbamylase, dihydroorotase, and repression of initiation factor 4E-binding protein 1 activity, which regulates nucleotide biogenesis and protein translation, respectively. Furthermore, inhibiting mTORC1 or PI3K inhibits viral replication and reactivation (89, 90). In another study, rapamycin unexpectedly failed to inhibit HIV reactivation in *in vitro* TCR-activated resting CD4^+^ T cells from individuals on suppressive ART. However, co-treatment of TCR-activated CD4^+^ T cells from ART-treated patients with rapamycin dramatically reduced cellular proliferation and secretion of pro-inflammatory cytokines, such as IL-2, TNF, and MIP1-α while reducing markers of T cell activation (CD25) and exhaustion (PD-1) and preserving basal CTL-mediated killing of infected cells (91). This provided an experimental model supporting a curative approach in which mTOR drugs could mitigate LRA-mediated inflammatory responses and toxicity while maximally reactivating the HIV reservoir. This provides new therapeutic opportunities to suppress homeostatic proliferation of key HIV reservoir cells and reverse latency (i.e., “starving the reservoir”) (14, 92). Indeed, genome-wide screening using *in vitro* models of HIV latency has identified the mTOR complex as a critical modulator of HIV latency (93). Thus, a combined approach modulating HIV reservoir metabolic machinery with or without cellular activators (to reverse latency) to limit pro-inflammatory cytokine production and homeostatic proliferation represents a novel chain of thought in HIV cure research (91).

## Metabolic Control of Monocytes in HIV

Like CD4^+^ T cells, monocytes contribute to chronic HIV infection (94). HIV-infected CD16^+^ monocytes have markedly greater Glut-1 expression, glucose uptake, and lactate production than CD14^+^CD16^−^ monocytes (95). HIV likely hijacks the glycolytic pathway of CD16^+^ monocytes for viral replication; however, confirmation of this, by inhibiting the glycolytic pathway, is needed. Notably, as the gut becomes “leaky” due to a loss of mucosal integrity, plasma lipopolysaccharide (LPS) is elevated in HIV positive patients (96), which may contribute to the increased activation of monocytes during HIV infection. This allows microbial products, such as LPS, to cross into the bloodstream, creating an inflammatory environment for circulating monocytes. Indeed, Glut-1^+^ monocytes express higher levels of TNF-α than Glut-1- monocytes, and the abundance of circulating Glut-1^+^ monocytes is strongly correlated with inflammation (95). Remarkably, increased Glut-1 expression on intermediate monocytes from HIV-infected women is associated with subclinical cardiovascular disease, suggesting a link between monocyte dysfunctional metabolism and inflammatory-mediated HIV-associated comorbidities (97). So far, the contribution of metabolic pathways in monocyte subsets to their specific functions has not been explored and requires investigation. The common theme emerging across the various leukocyte populations reveals that, in response to inflammatory stimuli, cells re-direct their metabolic preference to utilize glucose through the glycolytic pathway. What remains to be defined is the precise metabolic regulators that are induced to achieve this glycolytic state in monocytes, and how long this switch in metabolism lasts in these cells. In the context of LPS-activated monocytes, the high glycolytic rate is orchestrated by mTOR and HIF-1α, both known master regulators of glycolysis (51).

Thus, it will be important to determine if changing the metabolic phenotype of monocytes could alter their functional phenotypes in the blood, and if this affects the outcome of innate immune cell responses during HIV infection.

## Intervention Approaches That Modulate the Metabolic Profile and mTOR Activity in Immune Cells Could be Used to Fine Tune Their Development and Function

At present, a large body of evidence implicates metabolic pathways and their regulation by mTOR in governing development and function of Teff and Tm cells. One can, thus, envisage a potential benefit for interventions to direct the more efficient and optimal development of Teff versus Tm cells after an infection or vaccination. Some of the most obvious candidates are sirolimus (Rapamune) and everolimus (Certican), which are mTOR inhibitors and have been approved by the Food and Drug Administration as immunosuppressors for use in the context of organ transplantation (98). Other interesting candidates are metformin, which has been used in the treatment of type 2 diabetes and 2-DG (99–101). 2-deoxyglucose has been used for decades as a tracer in autoradigraphic and positron emission tomography (PET) imaging and was tested recently in pre-clinical trials in patients with solid tumors and epilepsy (99–101). However, further studies are needed to fully warrant the therapeutic use of 2-DG which has been shown to interfere with N-linked glycosylation of intracellular proteins in which glycans are added to the nitrogen of asparagine or arginine side chains (102). Because of its structural similarity to mannose which is essential in the glycosylation process, 2-DG effectively impedes N-glycosylation which is necessary for the proper folding of proteins (102). As a consequence, 2-DG may cause ER stress and lead to cellular apoptosis (102). On another front, accruing evidence suggests that ligation of PD-1 promotes FAO by enhancing expression of Cpt1a and inhibiting glycolysis in CD4^+^ T cells (103). Importantly, PD-1, which is highly expressed on exhausted CD8^+^ T cells, regulates their metabolic fitness during effector cell development (104). Blocking PD-1 *in vivo* or using PD-1-deficient LCMV-specific P14 cells reduced mitochondrial mass and increased glycolytic capacity in response to chronic clone 13 LCMV infection (104). Additionally, PD-1 deficiency correlated with a higher expression of the peroxisome proliferator-activated receptor γ coactivator 1α (PGC-1α), which regulates mitochondrial biogenesis and OXPHOS. This increase in PGC-1α correlated with reduced mitochondrial mass and enhanced glycolysis, yielding a more pronounced expansion and competitive fitness of PD-1-deficient, exhausted CD8^+^ T cells during the effector phase. Therefore, 2-DG and PD-1 ligation are potentially attractive targets for boosting FAO, which promotes Tm development and function.

Moreover, the cross talk between mTORC1 and mTORC2 is critical in regulating CD8^+^ Teff versus Tm cell fates *via* specific transcriptional reprogramming (105). Specific mTORC1 knock-down impairs mTORC2 activity and AKT-dependent activation (105). Interestingly, mTORC1 and mTORC2 regulate FOXO-1 *via* AKT-dependent phosphorylation. Rapamycin treatment impairs FOXO-1 phosphorylation, and this promotes its nuclear retention where it represses T-bet and drives the expression of Eomes, Tcf-1 transcription factors, and CPT1a, which collectively drives CD8^+^ Tm development (52, 105). On the other hand, higher mTORC1 activity leads to the phosphorylation and inactivation of FOXO-1 by promoting its nuclear export leading to reduced Eomes, but higher T-bet which promote CD8^+^ Teff differentiation (105). Additionally, inhibiting glycolysis using 2-DG also promotes the nuclear compartmentalization of FOXO-1(27). Therefore, inhibition of AKT or FOXO-1 phosphorylation is certainly additional avenues that warrant further investigation. On another front, mTORC1 activity is induced by amino-acid uptake, in particular, leucine which promotes mTOR localization to lysosomes that prompt mTOR activation. Inhibiting amino-acid transporters may be another way to modulate intracellular mTOR activity. Other key regulators of mTOR and metabolism are IL-2 and IL-15. Indeed, IL-2 tips the balance in favor of Teff instead of Tm development. In contrast, IL-15 is a key regulator of memory development that may occur in part through its capacity to enhance mitochondrial mass and SRC. Other signaling modulators downstream of IL-15, such as AMPK and STAT5, contribute to metabolic reprogramming (106). Thus, several attractive candidates exist that can serve as targets for novel therapeutic approaches to augment superior CD8^+^ Tm development and function.

## Concluding Remarks

The link between metabolic regulation and its downstream sequelae on the activation and function of the immune system has enormous immunotherapeutic potential. This begs the question of whether sugar or fat metabolism needs to be manipulated to promote more robust immune responses. Our review highlights that both sugar and fat metabolism are critical in orchestrating decisions relating to the function and fate of immune cells. Translating the knowledge from metabolic reprograming at the cellular level to the bedside, whether to treat acute or chronic viral infections or inflammatory diseases is yet to be established. One concern about using mTOR and PI3Kinase as immunotherapies is fear of toxicity and undesirable effects. However, glucose uptake by insulin-sensitive cells, such as adipocytes, is regulated by other glucose transporter isoforms (e.g., Glut-4), which are less responsive to inflammatory signals than Glut-1 (107). Thus, while these drugs may be explored for the “block and lock” (suppress HIV reactivation) or “starve” (inhibit homeostatic proliferation of the reservoir) HIV cure strategies, reversal of immune exhaustion through modulation of CD8^+^ metabolism may improve CTL-mediated killing of infected cells (91).

Further studies are needed to understand how metabolic reprogramming of one or several immune cell types can influence the functionality of other immune cells. Finally, future studies will need to investigate how to rheostatically modulate specific immune cells *in vivo* without causing any harm, and how these approaches should be timed with respect to boosting immune responses to vaccines.

## Author Contributions

HS, AM, ML, CG, SS, DF, and CP wrote the manuscript. HS and CP designed figures. SC provided overall critical evaluation and offered insightful suggestions to improve content. All edited the manuscript and approved the final version for submission.

## Conflict of Interest Statement

The authors declare that the research was conducted in the absence of any commercial or financial relationships that could be construed as a potential conflict of interest.
